# Why nature matters: A systematic review of intrinsic, instrumental, and relational values

**DOI:** 10.1093/biosci/biad109

**Published:** 2023-12-26

**Authors:** Austin Himes, Barbara Muraca, Christopher B Anderson, Simone Athayde, Thomas Beery, Mariana Cantú-Fernández, David González-Jiménez, Rachelle K Gould, A P Hejnowicz, Jasper Kenter, Dominic Lenzi, Ranjini Murali, Unai Pascual, Christopher Raymond, Annalie Ring, Kurt Russo, Aibek Samakov, Sanna Stålhammar, Henrik Thorén, Egleé Zent

**Affiliations:** Department of Forestry, Forest and Wildlife Research Center, Mississippi State University, Starkville, Mississippi, United States; Department of Philosophy, Environmental Studies Program, University of Oregon, Eugene, Oregon, United States; Instituto de Ciencias Polares, Ambiente y Recursos Naturales, Universidad Nacional de Tierra del Fuego and, Centro Austral de Investigaciones Cientificas, Consejo Nacional de Investigaciones Científicas y Técnicas, Ushuaia, Tierra del Fuego, Argentina; Department of Global and Sociocultural Studies, Kimberly Green Latin American and Caribbean Center, Florida International University, Miami, Florida, United States; School of Natural Science, Sustainable Multifunctional Landscapes, Kristianstad University, Kristianstad, Sweden; Instituto de Investigaciones en Ecosistemas y Sustentabilidad, Universidad Nacional Autónoma de México, Ciudad de Mexico, Mexico; Instituto de Investigaciones en Ecosistemas y Sustentabilidad, Universidad Nacional Autónoma de México, Ciudad de Mexico, also Ceiba Centro de Formación y Desarrollo, Oaxaca, Mexico; Rubenstein School of Environment and Natural Resources, University of Vermont, Burlington, Vermont, United States; Global Change Institute, School of Geosciences, University of Edinburgh, Scotland, United Kingdom; School of Engineering, Newcastle University, Newcastle upon Tyne, England, United Kingdom; Department of Environment and Geography, University of York, York, England, United Kingdom; Aberystwyth Business School, Aberystwyth University, Aberystwyth, Wales, United Kingdom; Ecologos Research Ltd, Aberystwyth; Department of Environment and Geography, University of York, York, England, United Kingdom; Department of Philosophy, University of Twente, Enschede, the Netherlands; Geography Department, Humboldt-Universitat zu Berlin, Berlin, Germany, Snow Leopard Trust, Seattle, Washington, United States; Terrestrial Ecosystems Research Line, Basque Centre for Climate Change, Leioa, and with the Ikerbasque Basque Foundation for Science, Bilbao, Spain, Centre for Development and Environment, University of Bern, Bern, Switzerland; Helsinki Institute of Sustainability Science; Ecosystems and Environment Research Program, Department of Economics and Management, University of Helsinki, Helsinki, Finland; Department of Philosophy, University of Oregon, Eugene, Oregon, United States; intertribal nonprofit organization Se'Si'Le, Eugene, Oregon, United States; Hydro Nation International Centre, Aberdeen, Scotland, United Kingdom; Department of Landscape Architecture, Swedish University of Agricultural Sciences, Uppsala, Sweden; Department of Philosophy, Lund University, Lund, Sweden; Lab Ecología Humana, Instituto Venezolano de Investigaciones Científicas, Caracas, Distrito Capital, Venezuela

**Keywords:** assessments, biodiversity, philosophy, policy, ethics, sustainability

## Abstract

In this article, we present results from a literature review of intrinsic, instrumental, and relational values of nature conducted for the Intergovernmental Science-Policy Platform on Biodiversity and Ecosystem Services, as part of the *Methodological Assessment of the Diverse Values and Valuations of Nature*. We identify the most frequently recurring meanings in the heterogeneous use of different value types and their association with worldviews and other key concepts. From frequent uses, we determine a core meaning for each value type, which is sufficiently inclusive to serve as an umbrella over different understandings in the literature and specific enough to help highlight its difference from the other types of values. Finally, we discuss convergences, overlapping areas, and fuzzy boundaries between different value types to facilitate dialogue, reduce misunderstandings, and improve the methods for valuation of nature's contributions to people, including ecosystem services, to inform policy and direct future research.

The ways individuals, communities, and societies express, embody, or articulate the importance of nature and people–nature relationships take many forms. This diversity has important implications for research, policy, and valuation around nature and nature's contributions to people (NCP), including ecosystem services (Anderson et al. 2022, IPBES 2022). Recent publications emphasize the need to focus on the multiple and diverse values of nature to achieve socially equitable and environmentally sustainable outcomes (Chan et al. 2016, Himes and Muraca 2018, Kenter et al. 2019, Köhler et al. 2019, Zafra-Calvo et al. 2020, IPBES 2022). Simultaneously, numerous international bodies have recognized this need. In this vein, the Intergovernmental Science-Policy Platform on Biodiversity and Ecosystem Services (IPBES) commissioned and approved the *Methodological Assessment of the Diverse Values and Valuations of Nature* (hereafter, the *values assessment*), in which the organization found that policy decisions have been largely based on a narrow set of market values of nature, underpinning the global biodiversity crisis. The values assessment concludes that identifying multiple values and incorporating them into policymaking provides leverage points for transformative change toward more just and sustainable futures, in line with Agenda 2030, the Kunming-Montreal Global Biodiversity Framework, and other multilateral agreements (CBD 2022, IPBES 2022, Pascual et al. 2023, United Nations 2023).

The values assessment proposes a typology to synthesize ways of conceptualizing the values of nature across diverse disciplines and knowledge systems (IPBES 2022). Accordingly, nature's values may be organized on the basis of four interrelated dimensions: worldviews and knowledge systems (ontologies and ways individuals or groups interpret, inhabit, and modify the world around them), broad values (life goals and guiding principles), specific values (opinions and judgments about the importance and meaning of something in specific contexts), and value indicators (the quantitative measures or qualitative descriptions of importance given to specific values; Raymond et al. 2023).

In the present article, we focus on the dimension of specific values, using the most common classification found in the academic literature: intrinsic, instrumental, and relational values (IPBES 2022). Within the wider values typology, specific values reflect how people, communities, and societies justify why and how nature and people–nature relationships are important to them. They represent historical contributions from, inter alia, environmental education, environmental ethics, conservation biology, and ecosystem services literatures. Researchers in these disciplines have sought to address the value of nature for its own sake, nature's benefit to people, and the value of noninstrumental and meaningful people–nature relationships (Díaz et al. 2015, Chan et al. 2016).

As part of the values assessment, we conducted a systematic literature review of these three specific value types to identify core meanings, trends, themes, disciplinary discrepancies, areas of convergence, and policy implications (see Anderson et al. 2022). Following the IPBES methods guidelines, the review process was documented and made publicly available in an annex to the values assessment (Muraca and Gould 2022), and its results are presented and discussed in the present article to (1) identify the most frequently recurring meanings in the heterogeneous use of different value types and their association with worldviews and other key concepts in the wider typology of values developed in the values assessment; (2) to determine a core meaning for each value type that is inclusive enough to serve as an umbrella over different uses in the literature and specific enough to help highlight its difference from the other types of values; and (3) to discuss convergences, overlapping areas, and fuzzy boundaries between different value types to facilitate dialogue, reduce misunderstandings, and improve methods for pluralistic valuation of NCP (including ecosystem services), inform policy, and direct future research.

## Surveying the literature

**Figure 1.**
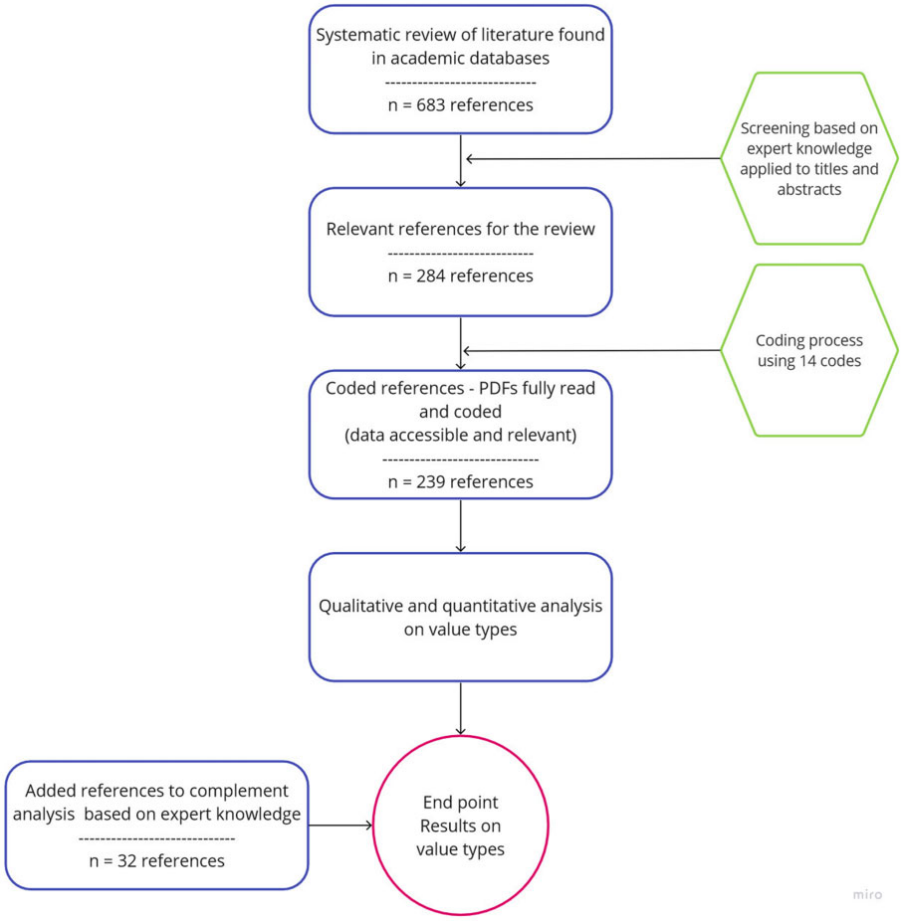
The process and workflow of systematic literature review for intrinsic, instrumental, and relational specific values. For further information and data management report, see Muraca and Gould (2022).

The literature review encompassed a systematized search, a qualitative analysis based on interpretive coding, and critical interpretive synthesis (figure 1; Dixon-Woods et al. 2006, Macura et al. 2019). The publicly available protocol guarantees the traceability and repeatability of the search process by documenting the search strings, the selection (inclusions and exclusions) criteria, and the interpretative codes used by all reviewers. We chose four academic databases (Web of Science, EBSCOhost Academic Search Premier, Google Scholar, and SCOPUS) to guarantee a wide spectrum of sources and to mitigate known disciplinary biases (Mongeon and Paul-Hus 2016). The searches were conducted in English in April 2020, with no limits on publication dates. The search terms in titles, keywords, abstracts, or subjects were adjusted according to the particular database structure and were focused on combinations of value terms (*intrinsic value, instrumental value, relational value*) with nature-related terms (*ecosystem services, nature's contributions to people*, or *nature*; see Muraca and Gould 2022 for details).

After a first selection of results (elimination of duplicates and articles not relevant to the topic on the basis of title and abstract), we identified 284 relevant articles for potential coding. We further eliminated materials for which we could not find full texts; the full text was written in a language that the reviewers could not understand; or, on reviewing the full text, the paper did not address values of nature—for example, if the term *nature* was used as synonymous of *essence* and had no reference to the environment.

After these eliminations, we coded 239 articles. The reviewers analyzed the literature on the basis of a shared codebook with categories relevant to the values assessment (see Muraca and Gould 2022). The coding was carried out as a form of qualitative content analysis; the majority of the codes entailed descriptive content as opposed to a predefined typology or data range.

For the interpretive critical analysis, the following five codes were analyzed: general information (the location of the study, the location of the first author's institution, and if the paper was an empirical work, a review, or a perspective); the worldviews directly or indirectly addressed (biocentric or ecocentric, strong anthropocentric, weak anthropocentric, pluricentric, or other, according to the IPBES values assessment; IPBES 2022); the ways in which people–nature relationships were otherwise expressed (e.g., connection to nature, human–nature relatedness, biocultural diversity, sacred landscapes); whether and which value types were explicitly or implicitly addressed (i.e., intrinsic, instrumental, relational) and how the value types were defined (verbatim quotes) or indirectly described or intended (verbatim quotes or paraphrase); and policy relevance (the impact on policy of multiple value types, including value pluralism).

Given the different history of use and variations of meanings of each value type, it was important to capture the implicit meanings of the value types. This was particularly relevant for relational values, which only began to be explicitly and broadly used in the general environmental literature in 2016. To represent implicit meanings, we referred to a tentative description of the semantic field of each value type (including different meanings) that was articulated collaboratively in common coding guidelines. These descriptions of value types were based on the present authors’ expert knowledge and validated through public and open reviews of early drafts of the values assessment according to the IPBES assessment process (www.ipbes.net/guide-production-assessments). To ensure quality control during the coding process, the codebook was constructed iteratively through discussions among the reviewers, whereby changes were made as emerging themes developed.

To ensure interreviewer reliability, the definitions and descriptions for all of the codes were shared among all of the reviewers, and we held meetings to discuss the process, answer questions, and ensure collective understanding of the coding process and goals. While they were coding, the reviewers were encouraged to record notes and additional points of interest for each paper. After coding, we performed an interpretive analysis of all notes and codes and a synthesis of the results. We developed the quantitative analysis of data ex post, exclusively for the present article and not as part of the values assessment.

Through interpretive analysis, we identified core meanings, salient articulations, and relevant associations with worldviews, broad values, and other relevant concepts (such as ecosystem services). We extracted the core meanings of intrinsic, instrumental, and relational values from the most frequent expressions used in the coded literature, with the goal of identifying umbrella definitions capable of covering significant variations of meanings while also highlighting differences between the value types. The core meanings we propose aim to collate a wide set of uses of each value type, which we call *salient articulations*, into operational definitions that can offer guidance, inter alia, for coding in valuation studies and empirical research. Salient articulations reflect the different ways in which the terms intrinsic, instrumental, and relational values are used in the literature and highlight different dimensions of meaning given to the specific value types. We limit the analysis to commonly used and sufficiently explained salient articulations. When a salient articulation for one value type overlapped with other types, we classified it according to the relative frequency and relevance with respect to the core meaning.

Finally, we assess patterns of relevant associations between the three specific value types and worldviews most frequently related to people–nature relationships as identified within the values assessment (i.e., anthropocentric, bio- or ecocentric, and pluricentric; Anderson et al. 2022). Relevant associations were noted when the reviewer interpreted that the paper aligned with a particular worldview and included explicit references to worldviews in quotes or by paraphrasing implicit references.

After coding, on noticing the absence of some seminal papers on environmental values (highly cited), coauthors and other contributors to the values assessment were asked to review the literature list and suggest, on the basis of their expert knowledge, relevant works that were missing (see the “Study limitations” section). As a result, 32 references were added. These papers were considered in the interpretive synthesis but not in the quantitative analysis.

We also realized that papers rooted in Indigenous and local knowledge (ILK) approaches were poorly represented in our data set. We collaborated with other experts within the values assessment, who had completed a parallel literature review on ILK, and invited them to complement the results of the interpretive synthesis by analyzing implicit expressions of the three value types in their data and offering correctives, comments, and examples (Athayde 2022).

## Findings from the literature

In this section, we present the results of the literature review, through a quantitative analysis of the data set, a qualitative analysis, and an interpretive critical synthesis of the coding. We do not engage in theoretical debates about the correct or inaccurate characterization of each value type but present its use in a comprehensive literature set. Like any qualitative interpretative analysis, validity is secured by the transparency of the process and the criteria used but does not happen in a vacuum. Choices about what is emphasized are influenced by the reviewers’ own positionality and values, albeit controlled by the process rigor. To secure transparency, the sources we cite in the results as specific examples of the core meanings, salient articulations, and relevant associations are from the literature search; we reference other sources where we believe they are necessary to provide additional context or clarifications. The full list of reviewed and coded literature is publicly available (https://zenodo.org/record/6499466).

### By the numbers

**Figure 2.**
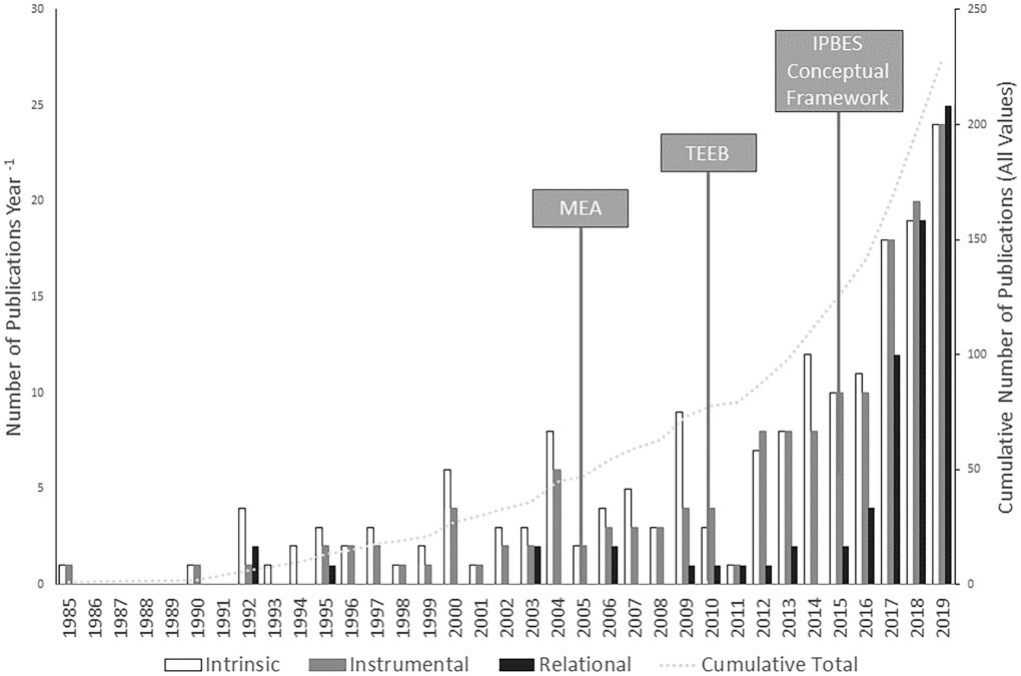
The annual number of publications from 1985 to 2019 that focus on specific values of nature. The callouts indicate pivotal framework publications, posited to affect research on the values of nature, the Millennium Ecosystem Assessment (MEA 2005), The Economics of Ecosystems and Biodiversity (TEEB 2010), and the Intergovernmental Science-Policy Platform on Biodiversity and Ecosystem Services’ conceptual framework (Díaz et al. 2015). Many of the papers referred to more than one value type, so the cumulative number of publications (the dashed line) is less than the sum of each specific value (the columns).

Since the first identified reference in 1985 in the searched databases (Førsund 1985), the number of publications on intrinsic, instrumental, and relational values has increased steadily over time. There is a marked increase in literature related to specific values in the early 2000s, coinciding with the work of the Millennium Ecosystem Assessment and the publication of the initial *Ecosystems and Human Well-Being* report (MEA 2005). Another period of growth in the 2010s corresponds to the publication of The Economics of Ecosystem and Biodiversity (TEEB) in 2010 and the initiation of IPBES (established in 2012, conceptual framework published in 2015; Dıaz et al. 2015). Although intrinsic and instrumental values were present in the literature across the whole period, the first explicit mention of relational values occurred in 2016, after the introduction of the IPBES conceptual framework (Díaz et al. 2015) and the foundational paper by Chan and colleagues (2016), which popularized the term and drew on the concept as presented in Muraca (2011), but these three papers were not recovered using our search criteria (see the “Study limitations” section). However, earlier papers implicitly evoked the concept of relational values. Intrinsic and instrumental values are most prevalent in early years; then the number of relational values publications catches up, contributing substantially to the overall increase in publications on the specific values of nature (figure 2). It should be noted that the use of relational categories to understand society– or community–nature relationships significantly predates the introduction of the term relational values, especially in sociology and anthropology (Emirbayer 1997, Viveiros De Castro 2004).

**Figure 3.**
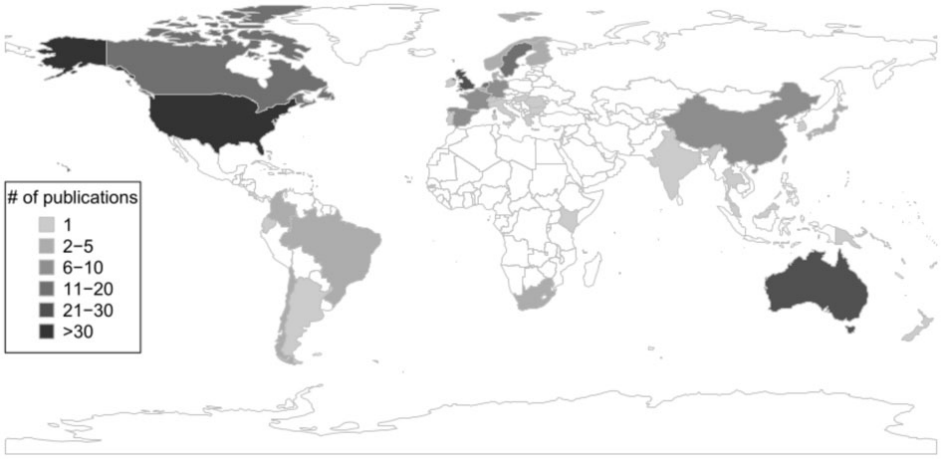
A map showing the geographic distribution of reviewed publications (*N* = 239) on intrinsic, instrumental, and relational values of nature; ecosystem services; and nature's contributions to people, based on the country of the first author's primary institution address. The United States had the largest number of publications (*n* = 63).

The publications came from first (lead) authors with affiliations in 40 different countries. The largest number were from the United States (63), followed by the United Kingdom (29), Australia (22), the Netherlands (16), Canada (16), and Sweden (12), with other countries represented by fewer than 10 publications (figure 3).

**Figure 4.**
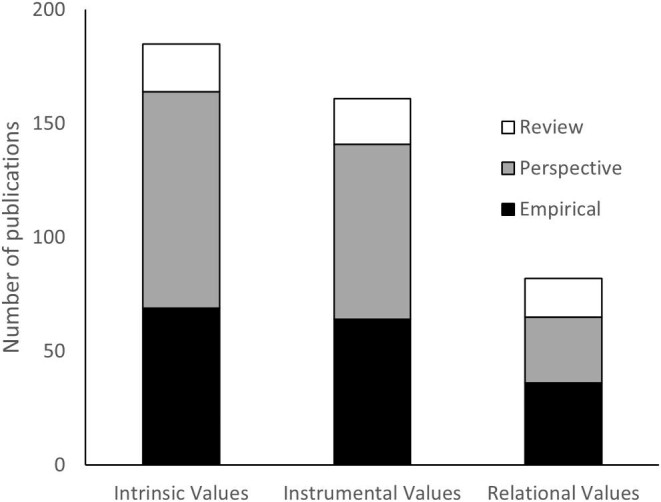
The number of reviewed publications that address intrinsic, instrumental, and relational values considering the contributions that were reviews, perspectives, or empirical studies for each value type.

The papers were classified as perspectives, including theoretical, conceptual, philosophical, and editorial pieces (46%); empirical studies (40%); and review articles (13%). Most of the papers referred to intrinsic (77%) or instrumental (67%) values. The publications focusing on relational values accounted for 34% of the reviewed papers. Although intrinsic and instrumental values had similar proportions of empirical (37% and 40%, respectively), perspective (51% and 48%, respectively), and review publications (11% and 12%, respectively), the relational values literature had a comparatively larger percentage of empirical (44%) and review (21%) articles with a corresponding lower percentage of perspectives (35%; figure 4).

### Intrinsic, instrumental, and relational values: Core meanings, salient articulations, and relevant associations

Table 1 summarizes the most relevant results from the qualitative analysis and interpretive critical synthesis, which classified each value type according to its core meaning, salient articulations, and relevant associations with worldviews and other concepts. Identifying a sufficiently distinct core meaning on the basis of relevant salient articulations for each value type on the ground of a review of interdisciplinary literature helps define each semantic field more clearly. It is also helpful for identifying categories and codes in valuations studies and empirical research and as reference basis for comparability across studies.

**Table 1.** Summary of core meanings, salient articulations, and most relevant associations of intrinsic, instrumental, and relational values that emerged from a systematic literature review and subsequent coding of 239 publications.

Value	Core meaning	Salient articulations	Relevant associations
Intrinsic	Values of entities expressed independently of any reference to people as valuers (including values associated with entities worth protecting as ends in and of themselves)	Defined negatively as noninstrumental value Value of something that is an end in itself, has agency Objective value or value independent of being valued or recognized by (human) valuer—inherent properties of something Regardless of importance or usefulness to humans Inherent moral value of natural beings (right to exist)	Strongly and explicitly associated with nonanthropocentric, biocentric or ecocentric worldviews Strongly associated with moral obligations toward other living things or life in general Weakly associated with biospheric and altruistic values, and with spirituality
Instrumental	Values of nature entities and other-than-human beings important as means to achieve human ends or satisfy human preferences (in principle replaceable, albeit not always in practice)	Means to an end (mostly intended as usefulness for humans, utility, or benefits, sometimes also for other-than-human beings) Leading to satisfaction of needs, preferences, interests, and desires Nature's value as a resource, for ecosystem services, as an asset, capital, or property	Strongly and explicitly associated with anthropocentrism Strongly and explicitly associated with utilitarianism and technocratic approaches to management
Relational	Values of meaningful and often reciprocal human relationships—beyond means to an end—with nature (often specified as a particular landscape, place, species, forest, etc.) and among people through nature	Values of or deriving from desirable, meaningful, just and reciprocal relationships with “nature” or between people through nature Values relative to or deriving from relationships that are constituent parts of identity (cultural, individual or collective) Values relative to or deriving from relationships that are constituent elements for living a “good life” Values associated with sense of place, including interconnection of cultural and sacred landscapes Values associated with care for or about specific landscapes, places, human and other-than-humans Value of nature as a point of connection among people, binding communities together and supporting social networks, such as in traditional markets	Strongly associated with relational, pluricentric or noncentric worldviews that question strict separation between nature and culture, society, or humanity and stress interdependence among all beings Strongly and explicitly associated with broad values, such as stewardship, responsibility, care, affection, reciprocity, harmony with nature, good life and justice Associated with cultural ecosystem services, as well as with spirituality

Note: The table also summarizes common associations of each value type with different worldviews, broad values, and other value-related concepts.

With respect to the relevant associations with worldviews, we generally followed the categorization of worldviews articulated in the values assessment values typology (IPBES 2022). Accordingly, biocentric and ecocentric worldviews were considered together, despite their differentiation in environmental ethics, because they share a nonanthropocentric perspective and can both be considered nature centered. Anthropocentrism is presented on a spectrum between weak or relational (recognizing human dependence on other beings) and strong or narrow (human superiority over other species) anthropocentrism. Pluricentric worldviews focus on a web of reciprocal and systemic relationships between human and other-than-human beings.

The term intrinsic value with reference to other-than-human beings is used in the literature with different, sometimes confused, meanings (O'Neill 1992, 1993). Intrinsic values are characterized as opposite to instrumental values, as the value of something that is an end in itself, as values independent of human judgment, as independent of human interests or well-being, and as the inherent moral value (in the sense of being a holder of rights) of other-than-human beings. In the IPBES conceptual framework, intrinsic values are equated to nonanthropocentric values and defined as the value of an entity independent of how it relates to humans (Pascual et al. 2017). In this section, we do not engage with theoretical discussions of appropriate or inappropriate uses and definitions but summarize and analyze the findings from the literature review.

Considering these differences and variations of use, we propose the following definition as an operational core meaning of *intrinsic value*: “values of other-than-human beings expressed independently of any reference to humans as valuers, including values associated with entities worth protecting as ends in and of themselves.” This definition serves as an umbrella meaning for most salient articulations by focusing on the justification behind them. Accordingly, expressing that other-than-human beings have intrinsic value does not necessarily mean that they have no relation to people (Sagoff 2009) but that the reason they are valued is explicitly expressed regardless of that relationship (Himes and Muraca 2018). This can include recognizing that nonhuman beings have their own interests and needs that warrant consideration (Rolston 1993, Sandler 2010, Berry et al. 2018). The definition is consistent with biocentric worldviews (King 2006, Batavia and Nelson 2017, Piccolo 2017) and aims at bridging subjective (people attributing intrinsic value to nature) and objective (value existing in nature regardless of people's attribution) understandings of value. To account for perspectives insisting on the objective nature of values, we added to the definition a reference to the understanding of intrinsic values as the value of entities that are worth protecting as ends in themselves. Framed this way, intrinsic values are not only assessed through biophysical indicators, such as abundance and endemism, but can also be subjectively articulated by people (Callicott 2002), who might act on them and acknowledge consequences to or rights for other-than-human nature (O'Connor and Kenter 2019).

We identified five salient articulations of intrinsic value (table 1). The first defines intrinsic values negatively as noninstrumental values (e.g., Weesie and van Andel 2008, Fürst 2015, Vucetich et al. 2015). This salient articulation is straightforward and often implicitly presupposed in literature on intrinsic values, but the negative definition has limited usefulness by itself unless a strict dualism between intrinsic and instrumental values is assumed.

The next salient articulation defines intrinsic values as the value of something that is an end in itself or has agency. Within this articulation, we include descriptions of nonhuman nature being valuable for its own sake (e.g., Lockwood 1999, Reyers et al. 2012).

Furthermore, intrinsic value is described as independent of being valued or recognized by a (human) valuer (e.g., Dion 2000, Hovardas 2013, Gale and Ednie 2019). This includes reference to inherent properties of an entity and to the objective value of nonhuman nature that exists regardless of human preferences, attitudes, or even their existence (Sheng et al. 2019). In the literature, this articulation is often presented in terms of nonanthropocentric values.

Intrinsic value is also articulated as the value of nonhuman nature regardless of its usefulness to humans or human well-being (e.g., Ghilarov 2000, Devos et al. 2019, Hugé et al. 2020). This understanding includes what is commonly known as *subjective intrinsic values*, which refers to values attributed by people to something that is valuable for its own sake to them and not for its usefulness; this category often includes aesthetic values (van Koppen 2000, Swift et al. 2004, Schröter et al. 2014).

Intrinsic value is used to address the inherent moral value of other-than-human beings, including arguments for nonhuman nature's rights to exist and other rights-based justifications (e.g., Alho 2008, Falk-Andersson et al. 2015, Sarkki et al. 2019). It resonates with biocentric conservation and some animal rights literature (Regan 1992, Rolston 1993, Batavia and Nelson 2017), which often imply moral obligations toward other-than-human entities (Schuler et al. 2017), and sometimes with the language of existence value intended as the right to exist regardless of function (Pearson 2016).

Intrinsic values are strongly and often explicitly associated with nonanthropocentric worldviews (Kahn 1997, Freemuth 2001, King 2006, Gilbert et al. 2009). This is not surprising, because most of the salient articulations of intrinsic value focus on the value of nature as independent or separate from humans or insist on the stand-alone value of other-than-human life.

Intrinsic values also tend to be associated with broad values that emphasize moral obligations toward nonhuman nature, other living things, or life in general (e.g., Harrop 2013, Gray and Curry 2016, Öhman et al. 2016), whether it be animals, species, all living beings, or ecosystems. Less commonly, intrinsic values were associated with sacred values, other-regarding, or biospheric broad values (Hattingh 2014, May 2017).

We propose an operational core meaning of instrumental value as “values of other-than-human entities, as means to achieve human ends or satisfy human preferences.” This core meaning includes “economic values, regardless of whether the entity is directly or indirectly used or not used” (Brondizio et al. 2019, p. 22). Accordingly, natural entities are important not in themselves but insofar as they provide (potential) utility to humans (Chan et al. 2016) or support communities’ economic well-being or subsistence (Lau et al. 2019, Hugé et al. 2020). This is expressed in many second-generation constitutions (i.e., constitutions that refer to social rights; Jung et al. 2014), which recognize people's right to a clean environment. Because instrumental values refers to a means to an end, the means might be substitutable (Schröter et al. 2020), at least in principle, even if not always in practice: That is, it is acceptable to consider equivalents or substitutes, if any are available or possible, that can provide similar benefits.

The core meaning represents the semantic field of instrumental values with a narrow focus related to preferences and utility, which is dominant in the literature we reviewed. Although the narrow focus might underrepresent broader understandings of instrumental values, it allows for a more specific characterization that helps distinguishing them more clearly from other value types. Broader understandings of instrumental values beyond the means–ends relation, referring, for example, to ecological functions or aesthetic values, tend to significantly overlap with the semantic field of relational and sometimes intrinsic values, which we discuss below in the “Fuzzy boundaries and overlapping meanings” section.

We identified three salient articulations, often overlapping, of instrumental values. The first refers to the value of other-than-human nature as means to an end (e.g., Lockwood 1999, Reyers et al. 2012, James 2020). In most cases, the end is intended as usefulness, utility, or benefits, for humans, although some scholars also stress the instrumental value of something as a means for ends set by other-than-human beings (Piccolo 2017).

The second refers to the satisfaction of needs, preferences, interests, or desires (e.g., Öhman et al. 2016, Jones and Tobin 2018, Gale and Ednie 2019). The papers using this salient articulation sometimes refer to nonuse benefits of nature, usually referencing the total economic value (TEV) classification (TEEB 2010), including altruistic, bequest, or existence value types (Hattingh 2014, Farrell et al. 2017, Christie et al. 2019).

The last salient articulation refers to nature's value as a resource for the delivery of ecosystem services, as an asset, capital, or property (e.g., Beltrani 1997, Bonnett 2012, BenDor et al. 2014, Batavia et al. 2018, Berry et al. 2018). This includes reference to the importance of sustainable use and environmental policy to maintain or enhance natural capital. Currently, this understanding is best articulated in TEEB (2010) and by the recent Dasgupta review, in which nature is defined as an asset (Dasgupta 2021).

Instrumental values are strongly and explicitly associated with ecosystem services and anthropocentric worldviews (Kahn 1997, Reyers et al. 2012, Hovardas 2013). In almost all cases, the ends of instrumental values and the beneficiary of nature's resources or services was human (e.g., Winter and Lockwood 2004, Pelenc et al. 2013, Bremer et al. 2018). Instrumental values also tended to be strongly and explicitly associated with utilitarianism and paradigms of managing nature (Alho 2008, Falk-Andersson et al. 2015, Farrell et al. 2017).

Given the more recent history of the use of relational values as a specific value in environmental literature, different meanings and uses of the term coexist. There is ongoing debate whether they are a separate type of value (Norton and Sanbeg 2021, James 2022, Luque-Lora 2023, Piccolo et al. 2022) or whether they should be considered as a boundary object (Stålhammar and Thorén 2019). We do not engage in the debate in the present article, but rather focus on how relational values are presented in the reviewed literature. The term is often used in the literature to express the value of noninstrumental human–nature relationships or emphasize relationships that are, in principle, not substitutable and lose their meaning if translated into narrowly instrumental language (Jax et al. 2013, Arias-Arévalo et al. 2017, Klain et al. 2017, Chan et al. 2018, Himes and Muraca 2018), as in the case of friendship, which is important precisely because of the relationship but loses its meaning if reduced to a means to an end (O'Neill et al. 2008). The language of intrinsic values is generally not helpful to articulate relational values, because most framings of intrinsic values explicitly disregard relationships in the justification of importance.

We propose the core meaning of relational values as the “values of meaningful, and often reciprocal human relationships—beyond means to an end—with nature and among people through nature, where *nature* is often specified as a particular landscape, place, species, forest, etc.” (Chan et al. 2016, Chan et al. 2018, De Vos et al. 2018, Himes and Muraca 2018, Schröter et al. 2020). Relational values are frequently framed as context dependent, often place-based, nontradable, and therefore largely not substitutable in principle (Kenter et al. 2019). They refer to complex human–nature relationships that are integral to a good quality of life and are important for how some people understand themselves as living in and through reciprocal relationships of responsibility in the bioculturally diverse world they inhabit (McGregor 2010, Kimmerer 2011).

We identified six salient articulations for relational values—the relatively recent and still open discussion of relational values justifies a larger variety of expressions and a lower level of synthesis in the use of the term.

First, close to the core meaning, as it was used more or less explicitly in the majority of the analyzed papers, relational values are intended as the values of or deriving from desirable, meaningful, just, and reciprocal relationships of people with nature and among people through nature (Chan et al. 2016, Schröter et al. 2020). The term often overlaps with other salient articulations that emphasize more specific types of relationships and is frequently evoked by citing the foundational Chan and colleagues (2016) paper or the IPBES framework's definition of relational values.

Second, relational values refers to values relative to or deriving from relationships that are constituent parts of people's identity (cultural, individual or collective; Musschenga 2004, De Vos et al. 2018, Gould et al. 2019). This articulation is helpful in expressing the value of people–nature relationships for indigenous peoples and local communities (IPLC). For example, in the New Zealand agreement between the Indigenous Whanganui Iwi (Māori) people and the Crown, the river Te Awa Tupua is acknowledged as connected with the identity of the iwi and hapū in an inalienable way, because the document literally says, “I am the River and the river is me” (Te Awa Tupua (Whanganui River Claims Settlement) Act 2017 2017).

Third, relational values refers to values relative to or deriving from relationships that are constituent elements for living a good life. This includes relationships with people and nature that are essential components of a meaningful and flourishing life (eudaimonia), worthy of a human being, including virtues and attitudes of responsibility (Klain et al. 2017, Jax et al. 2018, Schröter et al. 2020). For instance, Knippenberg and colleagues (2018, p. 43) insisted that “good relations are key constituents of the good life” and propose the concept of nature-inclusive eudaimonia, in which nature is considered constitutive of human flourishing.

Fourth, relational values are associated with sense of place (De Vos et al. 2018, Marshall et al. 2019, Skubel et al. 2019, Basu et al. 2020) and interconnected with cultural and sacred landscapes (e.g., Jax et al. 2018, Köhler et al. 2019, Sarkki et al. 2019). Examples include plural valuations of nature in protected areas (e.g., De Vos et al. 2018, Mrotek et al. 2019), values that motivate preservation of a specific landscape such as the sense of pride reported by Calcagni and colleagues (2019) by citizens of Chattanooga that builds connections between people and their city through a unique sense of place and culture. This articulation also includes landscapes that are sacred or have spiritual meaning such as *wakas*, or sacred sanctuaries, of Andean peoples, which are places for connection and renewal (May 2017).

Fifth, values associated with care for or about specific landscapes, places, human and nonhuman others, including values of responsibility and reciprocity (Gould et al. 2018, Jax et al. 2018, De Vreese et al. 2019), such as reciprocal responsibilities of giving and receiving between people and the natural world (May 2017, Norgaard et al. 2017). For example, in South America's Quechua language, reciprocity, or *ayni*, is the glue that holds everything together (May 2017), and in Hawaii, *e mālama i ka ’aina* means “take care of the land” (Gould et al. 2019). In northern California, for Karuk fishers, the “responsibilities to the natural world include ceremonial management of the fishery to ensure ‘escapement’ and burning of the forest to enhance runoff” (Norgaard et al. 2017, p. 103).

The final salient articulation of relational values refers to values of nature as a point of connection among people, binding communities together and supporting social networks (e.g., Norgaard et al. 2017, García-Llorente et al. 2018, Skubel et al. 2019). Many papers that fit this salient articulation reference Pascual and colleagues’ (2017, p. 12) assertion that “relational values reflect elements of… social cohesion,” but common and more detailed accounts are found in ILK literature; for example, Skubel and colleagues (2019) described how the Rrumburryia clan of the Yanyuwa people in northern Australia tell a story of “The Tiger Shark (*Ngurdrungurdu*) Dreaming,” which exemplifies how sharks are part of what binds humans and other-than-human nature together, or the *agdal* system, a traditional Berber form of environmental management in North Africa in which reciprocal relationships with the natural world are essential for supporting community cohesion, cultural coherence, and social networks (Dominguez et al. 2012). This articulation is also evident in intergenerational connections made through relationships to farming a place and farming as a way of life identified in interview responses of farmers in the US Northwest (Chapman et al. 2019).

Relational values are very strongly associated with pluricentric worldviews, which question the strict separation between nature and culture, society, or humanity and stress the interdependence between all beings (May 2017, Saxena et al. 2018, Devos et al. 2019, Gould et al. 2019). They are also very strongly associated with broad values of stewardship, responsibility, care, affection, reciprocity, harmony with nature, good life, and justice (Gudynas and Acosta 2011, De Vreese et al. 2019). Finally, relational values are also associated with cultural ecosystem services and spirituality (e.g., Harrop 2013, Hofstra 2017, Köhler et al. 2019).

### Fuzzy boundaries and overlapping meanings

Identifying a core meaning and salient articulations for each of the specific values helps distinguish them more clearly. This is useful for analytic reasons, such as developing questionnaires or coding criteria for qualitative research and literature review or defining categories to include in valuations of NCP. In some cases, a more accurate definition can clarify the differences among value types and enable a clearer identification of individuals or groups stressing different justifications of values and potential lines of conflict. At the same time, crosscutting meanings of the three value types are not to be quickly dismissed as inaccurate or vague and can bear significant relevance for research and policy by revealing the importance of context and perspectives rooted in diverse knowledge systems.

We found, in some contexts, that the meanings of intrinsic, instrumental, and relational values are contested and may overlap (Pascual et al. 2017, Himes and Muraca 2018, Schröter et al. 2020), creating fuzzy boundaries. For instance, we found—not surprisingly—overlapping meanings between relational and instrumental values with respect to material NCP such as food, which may have instrumental and relational value, depending on the context and local practices. For example, in Mahahe, wild fruit groves are appreciated instrumentally, for the important additions to the diet and the shade they offer, as well as relationally, as a gathering place for communing with each other and nature (Schnegg et al. 2014). These wild fruit groves simultaneously have instrumental and relational values to the Mahahe. Identifying fuzzy boundaries helps articulate the full measure of their importance to the community, which can otherwise not be adequately expressed by a single value type. At the same time, being able to analytically distinguish between instrumental and relational value articulations can help identify or monitor shifts in how the community understands their relationships with the trees or differentiate value articulations by age or economic status.

Justifications based on instrumental and intrinsic values often overlap when sentient animals are seen as ends in themselves and reducing their suffering could be justified under a utilitarian framework as instrumentally good for them (Rolston 1993, King 2006, Harrop 2013). In other instances, relational and subjective intrinsic value (something is important *for someone* for its own sake) or intrinsic value defined negatively as noninstrumental might be hard to distinguish. In other cases, fuzzy boundaries extend to all three value types, as with the sense of place. In many cases, relational values are equated with values of specific places (Devos et al. 2019) or a sense of place (Skubel et al. 2019); in other cases, intrinsic (Gruen 2002, Bonnett 2012), instrumental (Runhaar et al. 2019), or both types of values (Blennow et al. 2019) are attributed to the importance of place. The literature suggests that values can be socially or symbolically constructed through relationships with others in places (relational values), the sense of place can also be associated with the material properties of places (instrumental meanings) or the intangible emotional, symbolic, and spiritual meanings of places (expressed as relational or subjective intrinsic values; Raymond et al. 2010, Williams 2014).

**Figure 5.**
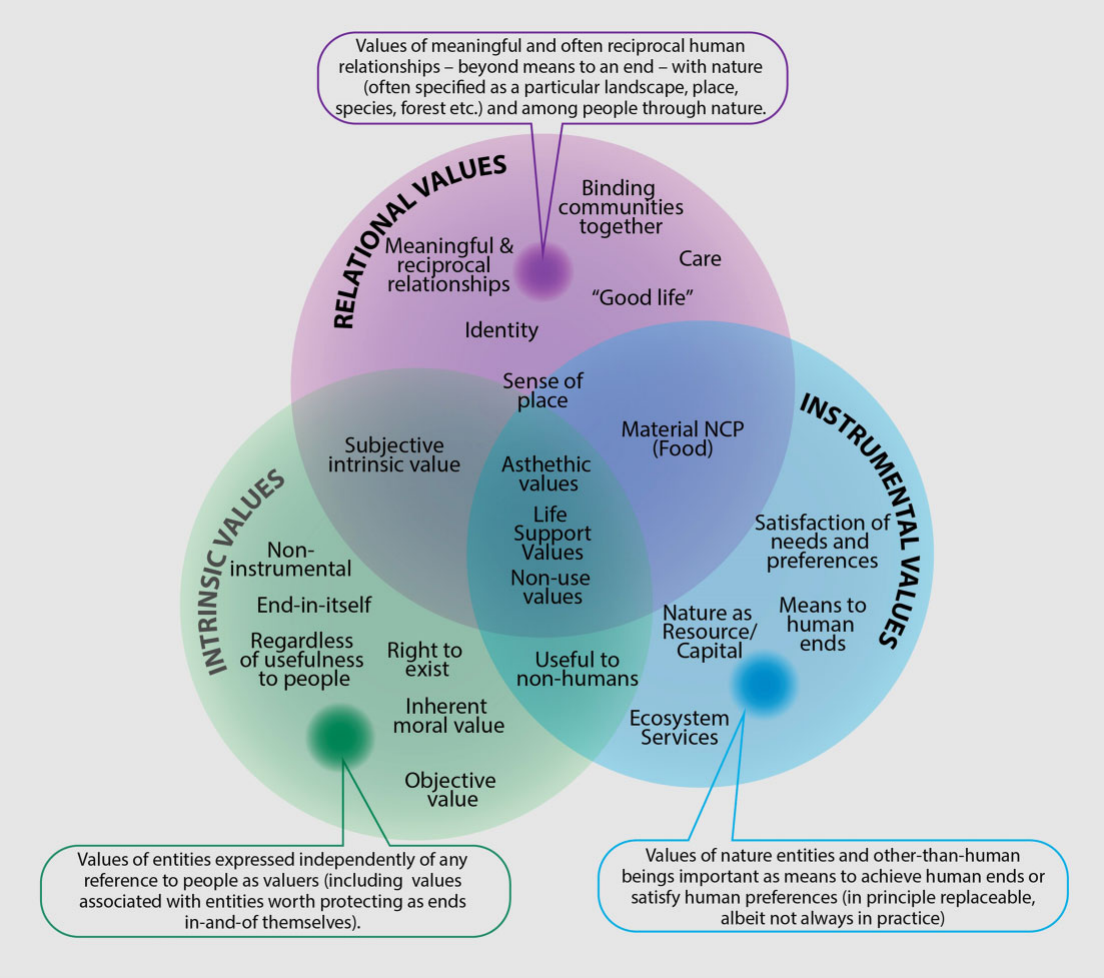
The categories of intrinsic, instrumental, and relational values may not adequately explain all values, and all three are underpinned by life-support values. The different *core meanings* are represented as layers or dimensions of each value type to illustrate the different ways each value type is represented in the literature and emphasize that the *core meanings* are not mutually exclusive categories but overlapping aspects of each value concept. Different types of specific values span value types; for instance, aesthetic values are described in the literature using all three specific value types.

In the remainder of this section, we discuss in more detail three fuzzy boundaries that we identified repeatedly where all three value types converged: nonuse values, aesthetic values, and values linked to life support processes, which we term *life-support values* (figure 5). We draw occasionally on additional literature from the value assessment besides the data collected for the systematic literature search in order to clarify concepts, introduce general themes, or support explanations with additional, relevant examples. Although the evidence of overlapping use of different value types was evident in the literature, indicating the existence of these fuzzy boundaries, the discussion of reasons for the fuzzy boundaries and potential significance for policy, practice, and decision-making results from the author's interpretation of the findings.

The term *nonuse values* originated in the economic literature and is distinguished from *use values*, as in the TEV framework. *Use values* refers to the satisfaction generated by the direct (consumptive or nonconsumptive) or indirect (the conditions that enable use or satisfaction) use of ecosystem services or NCP. *Nonuse values* “are based on the preference for components of nature's existence without the valuer using or experiencing it and are of three types: existence value, altruistic value, and bequest value” (Pascual et al. 2017, pp. 11–12). Besides this specific economic meaning, the term *nonuse values* is employed to articulate some instrumental values that cannot be represented straightforwardly in monetary terms or via market exchanges—for example, the rights of future generations to biodiversity or nature components (Winter and Lockwood 2004, Haggan 2011). Nonuse values are also sometimes evoked to express intrinsic values generally (Swift et al. 2004) or as synonymous with existence value (Buijs 2009, Zhang et al. 2013), and we identified implicit references to relational values in descriptions of nonuse values—for example, with reference to altruistic values (More et al. 1996, Pearson 2016).

From a theoretical point of view, interpreting existence value or altruistic value in terms of intrinsic or relational justifications results from a misinterpretation of the economic language (Kenter et al. 2015). Framing intrinsic and relational values in terms of TEV nonuse values might have consequences in terms of environmental and epistemic justice or might fail to adequately represent the complexity of environmental conflicts (Martinez-Alier 2002), leading to inadequate policies to address them (Anderson et al. 2022). For example, people generally perceive intrinsic values and many relational values as nonnegotiable and reject their reduction to the language of preferences, leading to environmental conflicts (Temper 2019).

**Figure 6.**
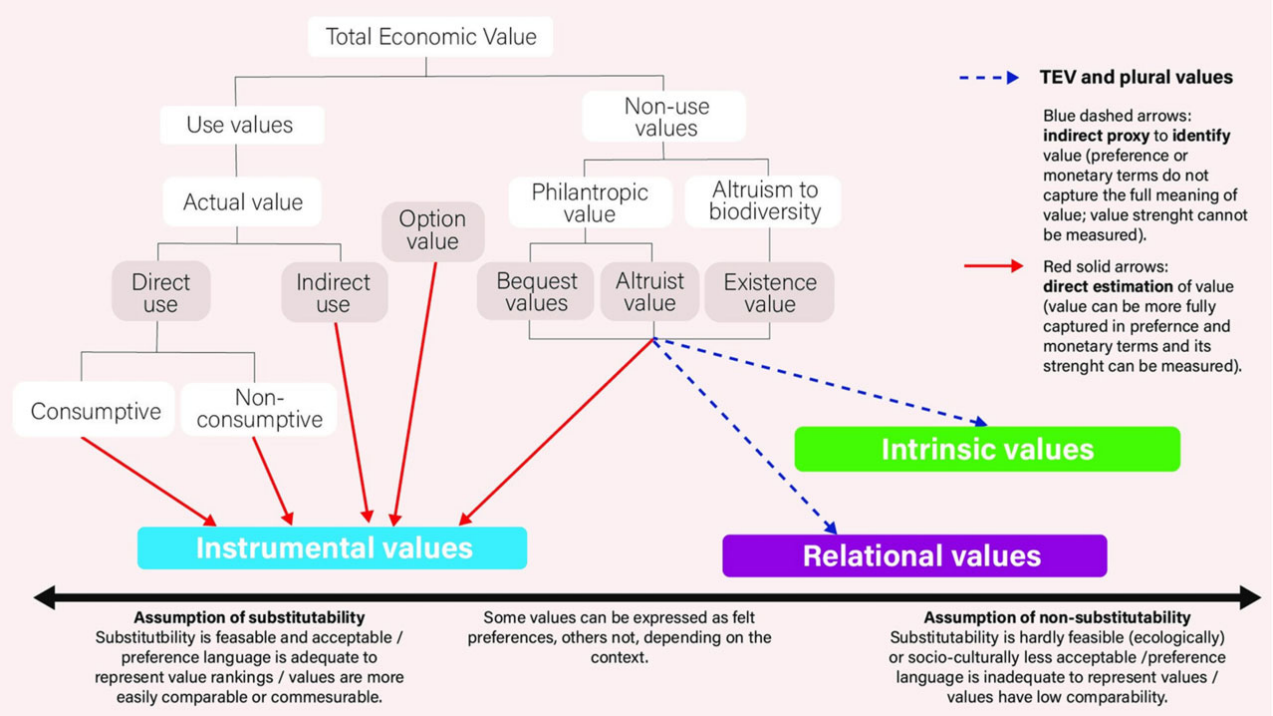
The total economic value classification framework encompasses multiple environmental value types. The figure presents a spectrum between stronger and weaker assumptions of substitutability between the objects of value. Source: The figure was adapted from the values assessment's chapter 2 (Anderson et al. 2022).

However, the less specific uses that occur in the literature may help identify instances when multiple values are at play and highlight attempts at finding a common language across groups (see box 1). Moreover, nonuse values, within limitations, may serve as indicators for when intrinsic or relational values are present but likely cannot be used to assess the full meaning of those values without complimentary, noneconomic indicators (see figure 6 in box 1).

Box 1. **How the total economic value classification of nonuse values relates to instrumental, intrinsic, and relational values.**

Total economic value (TEV) is based on a utilitarian, preference-based understanding of value that represents nonuse values in terms of the satisfaction generated for an individual by knowing that others will have access to nature's benefits, be it current (altruist value) or future generations (bequest value), or by knowing that something exists, even if there is no direct access to or direct enjoyment of it (existence value; Hansjürgens 2014, Anderson et al. 2022). The focus on preferences is mostly anthropocentric and instrumental, where value is assigned to biodiversity or ecosystem services “to the extent that these fulfill needs or confer satisfaction to humans either directly or indirectly” (TEEB 2010, p. 187). This implies that existence, bequest, and altruistic values are represented according to an instrumental value justification that allows for trade-offs, commensurability, and potential substitutability across the objects of value (Kenter et al. 2015, Anderson et al. 2022). As acknowledged by TEEB itself, nonuse values present “greater challenges for valuation than do use values since nonuse values are related to moral, religious or aesthetic properties, for which markets usually do not exist” (TEEB 2010, p. 196).

Although the TEV approach aims to capture instrumental values, other value types sometimes can be indirectly identified by framing them in the language of preferences (see figure 6). By borrowing language from Schröter and colleagues (2020), who employ socioecological indicators as proxies for relational values, we propose, in a similar vein, to use, when legitimate and within limitations, TEV categories as indirect proxies that can help identify that a preference for a value is present but cannot estimate the strength of that preference compared to others, nor can they be accurately used to assess the full meaning of that value. In these cases, noneconomic indicators should be added to replace TEV to better address environmental conflicts, and to support epistemic and recognition justice.

Aesthetic values are also addressed under all three categories in the literature. In terms of intrinsic value, the beauty of nature, a place, or an other-than-human entity is considered valuable for its own sake regardless and independently of usefulness to people and it is nonnegotiable (van Koppen 2000, Swift et al. 2004, Schröter et al. 2014, Marshall et al. 2019). In terms of instrumental value, beauty is conceived as a preference for a beautiful state of affairs over a different less beautiful state or because it causes aesthetic pleasure and can be expressed as willingness to pay or via hedonic valuation (the value of real estate in the vicinity of ‘beautiful’ green areas; van der Ploeg et al. 2011, Winter 2017).

In papers explicitly using the relational value concept, aesthetic values are defined as relational and noninstrumental; beauty is understood in terms of a relation to a specific place, landscape, ecosystem, or species that deeply informs the identity of an individual or community and their sense of belonging or willingness to care for that place (i.e., aesthetic experience is considered as an essential component of a good life; Muraca 2011, Saner and Bordt 2016, Schröter et al. 2020). Implicit references to relational values include, in our interpretation, the understanding of aesthetic appreciation as connected to sympathy toward and living in harmony with nature (Gao 2016). In this sense, articulating beauty only in terms preferences and trade-offs between them is firmly resisted, and the importance of the relation between valuer and valued object is highlighted (Deplazes-Zemp and Chapman 2021). Instead of considering this fuzzy boundary as a problem requiring a more precise or “right” articulation of aesthetic values, embracing the fuzziness can reinforce the importance of aesthetics and beauty as common ground across groups using different justifications. This common ground can be leveraged for the protection of biodiversity and ecosystems.

**Figure 7.**
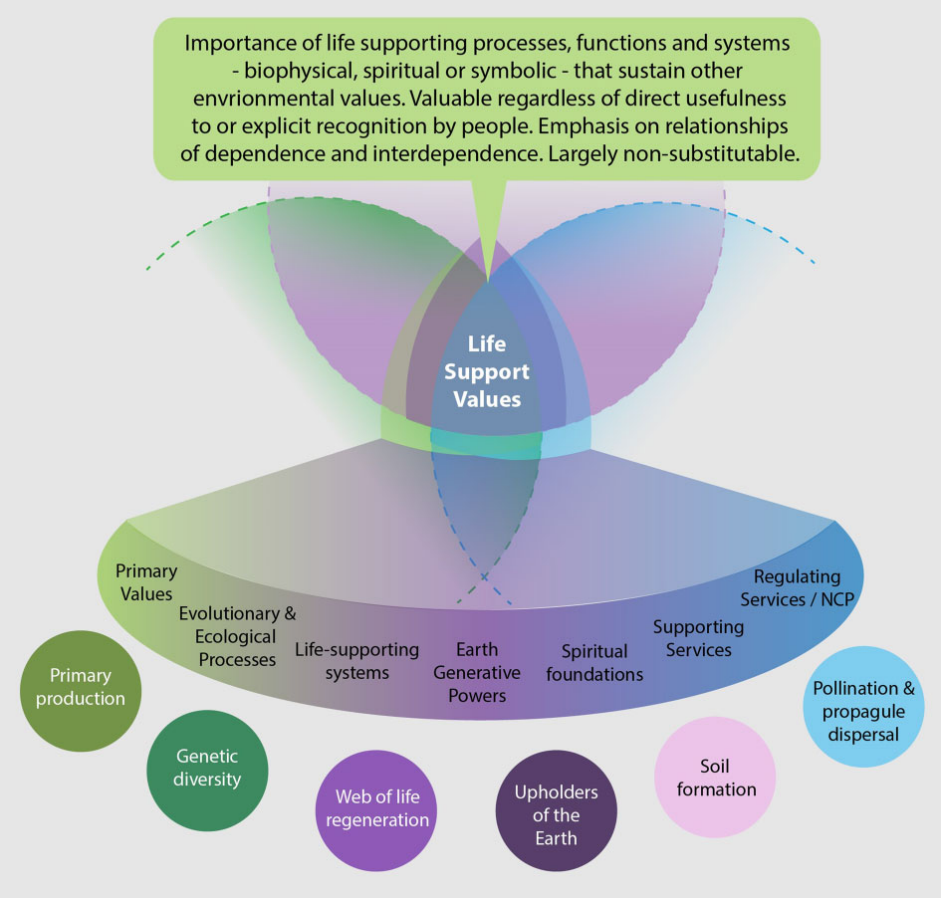
Fundamental values of nature. Those more associated with intrinsic values to the left, relational values in the center and instrumental values to the right.

The value of life-supporting processes, functions, and systems—interrelating biophysical, spiritual, or symbolic aspects—and relationships of dependence and interdependence with respect to them was also expressed in terms of all three value types. To account for these concepts found in the literature under the frame of intrinsic, instrumental, or relational values, we introduce the operational term *life-support values* (figure 7).

Within the semantic field of each value type, life-support values are largely described as not substitutable and foundational for other environmental values. Under intrinsic values, life-support values are framed in the literature as the importance of evolutionary and ecological processes that are independent of people's judgments, including the Earth system as a whole (Rolston 1993, Kahn 1997, Pelenc et al. 2013, Hattingh 2014, Fritz-Vietta 2016, Piccolo 2017), which enable other values (Rolston 1988). Under instrumental values, life-support values are framed in terms of ecological functions or as the value of the biotic and abiotic prerequisites for the functional reliability and the self-organization of the ecological systems and apply to the importance of supporting ecosystem services (Rolston 1993, Ghilarov 2000, MEA 2005, Farnsworth et al. 2012, Bottazzi et al. 2018), indirect use values or primary values (Hansjürgens 2014, Fritz-Vietta 2016), functional values (Lockwood 1999), critical natural capital (Battistoni 2017), and regulating NCP (Díaz et al. 2015). Under relational values, life-support values are presented in terms of fundamental values (Muraca 2011, Arias-Arévalo et al. 2018, Schröter et al. 2020) that express the importance of life-supporting processes that give sense to people's existence and identity. The latter is not limited to biophysical aspects but also includes the spiritual and symbolic meaning of life-giving and life-regenerating processes in specific contexts (e.g., contextual NCP), including, with reference to biophilia, “innate and beneficial connections with nature” (Ross et al. 2018, p. 47) or in terms of lifeworlds (Reis Cunha 2017). Examples include the Andean Indigenous concept of Pachamama, referring to Earth's generative powers and to the very constitution of life (Silverblatt 1987, Pacari 2009, Macas 2010, Tola 2018) and the contextual spiritual foundations for the regeneration of life, practices, and reciprocal relations the Dongria people express for India's Niyamgiri Mountains, which “not only provide the people with life and livelihoods, [but] they are also worshiped as the upholders of the Earth and the laws of the Universe” (Supreme Court of India 1995).

## Reflections on policy, research, and values communication

We now turn to discussing how we believe our findings may help facilitate dialogue, reduce misunderstanding, improve valuation of ecosystem services and NCP, inform policy, and direct future research. Below, we discuss how differences between and within value types are illustrative of historical development of value terms across divergent disciplines. We suggest that core meanings, salient articulations, and relevant associations illustrate some of these disciplinary discrepancies and may serve as an interpretive key to help interdisciplinary researchers and decision makers avoid confusion and communicate more clearly. We describe the strengths and limitations of each value type for describing why nature matters to people and argue that value pluralism guided by core meanings and salient articulations has important implications for ecosystem accounting at multiple scales (e.g., countries and industries who will now be responsible for creating ecosystem accounts as part of the Kunming-Montreal Global Biodiversity Framework). We suggest that fuzzy boundaries between value types are a natural starting point for pluralistic valuation. We conclude the section with study limitations and suggestions for how the core meaning, salient articulations, relevant associations, and fuzzy boundaries we identified can guide future research.

### The three value types have distinct histories and associations that are clarified by core meanings, salient articulations, and relevant associations

The histories of the three specific values of nature are different. They have distinct and partly overlapping trajectories. This has practical significance for how value concepts are used and defined in sustainability science and policy. Value terms need to be contextualized with regards to a given scholarly trajectory, which will have its own set of assumptions. Untangling these assumptions and intertwined trajectories is made easier by identifying the salient articulations of each value type being used and the relevant associations adopted in different disciplines and context.

In earlier science–policy interface documents, such as the MEA, mainly intrinsic and instrumental values are presented and typically depicted in dichotomic opposition (something may either have dignity—intrinsic value—or a price—instrumental value; MEA 2005). This dichotomy is represented in the salient articulation of intrinsic values defined negatively as noninstrumental value but can be further mapped onto two predominant approaches in the general environmental discourse. For instance, the fields of conservation biology and environmental ethics both invoke salient articulations of intrinsic values as the value of natural processes and systems “regardless of importance or usefulness to humans” and “the inherent moral value of natural beings (right to exist).” With the introduction of the CBD and the ecosystem services framework, instrumental (and relational) language has become more relevant in the debate (Norton 1991, Justus et al. 2009, Sagoff 2009, Batavia and Nelson 2017). In the sustainability discourse and in environmental and ecological economics, the language of instrumental value is increasingly dominant, primarily emphasizing the salient articulation of nature's value as a resource for ecosystem services, as an asset, capital, or property (Daily 1997, TEEB 2010).

However, despite being used in opposition, we found that the definitions of intrinsic and instrumental values sometimes overlap. Before the introduction of relational values to the environmental literature, many salient articulations of relational values would be designated confusingly as both intrinsic and instrumental. Since their introduction, relational values helped clarify the meaning and scope of environmental values in areas where instrumental and intrinsic value definitions overlapped, were inconsistent, or were not very clear, as is the case with identity-constituting relationships or social cohesion. Giving an explicit name to these values made them more visible and facilitated empirical research and assessments needed for policy (Christie et al. 2019, De Vreese et al. 2019, Chapman et al. 2020). The addition of relational values, to articulate the importance of noninstrumental relationships with nature and as a distinct value types (Muraca 2011, Chan et al. 2016), can mitigate confusing uses of intrinsic and instrumental values but only if scholars are willing to adopt it in their interpretation of literature predating the widespread use of the term relational values. This can be done more easily by keying in on salient articulations and relevant associations of relational values in earlier literature as indicators and evidence of implied relational values (e.g., values associated with spiritual meaning and the importance of caring and reciprocal relationships with nature). Recognizing relational values in earlier literature becomes more important as recent trends in the literature signal greater interest by empirical researchers to engage with them.

The success of relational values in valuation studies might also have indirectly contributed to narrowing down the semantic domain of instrumental values. Although from a theoretical point of view this might be contentious, being able to distinguish instrumental and relational domains in practice can improve the implementation of environmental policy affecting diverse communities (Lliso et al. 2022).

### Implication of value pluralism guided by the core meanings and fuzzy boundaries for policy and valuation of NCP

Finding appropriate language to represent the diverse values of NCP and ecosystem services has important policy ramifications (Campagna et al. 2017). We believe that each value type provides a distinct and important mode of communicating and justifying the importance of nature and people–nature relationships. By isolating specific core meanings from the literature, the relevance and limitations of each value type for policy and valuation of ecosystem services and NCP can be more clearly identified and different trajectories of values enquiry clarified. Moreover, fuzzy boundaries between different value types, once they are identified, are logical areas to find or build common ground between parties with different conceptualizations of value or resource management interests (Raymond et al. 2023).

Intrinsic values, as they are defined by the proposed core meaning in the present article, are considered, as we found in the reviewed literature, essential in environmental policy to sustain and trigger people's motivation for conservation (Polasky et al. 2012, Batavia and Nelson 2017), in education (Zhang et al. 2013), and to articulate the agency of other-than-human beings as expressed, for example, by Quechua communities in Peru about the mountain Ausangate as a powerful earth being (De La Cadena 2010). Intrinsic values are also closely associated with biocentric and ecocentric worldviews that continue to be important conceptualizations of nature in support of conservation. Appealing to intrinsic values can help legitimize environmental protections and improve policy success but may sometimes lack consideration of pragmatic elements relevant to environmental management (Minteer et al. 2004, O'Connor and Kenter 2019) or may disregard relational frameworks connecting people and land (Chapman et al. 2019).

Instrumental values, as they are defined according to the proposed core meaning in the present article, lend themselves, as we found in the literature, to quantitative analysis favored in valuation of ecosystem services and material NCP or resource management planning for sustainable development. Because they are substitutable in principle, they support high comparability and commensurability, which facilitates trade-off assessments that can be articulated in monetary units—for example, by adopting cost–benefit analysis or contingent valuation (Larréré and Larrére 2007). However, narrowly instrumental approaches to valuation that only consider, for example, monetary values may obscure other value expressions, crowd out other reasons for environmental protection (Rico García-Amado et al. 2013), alienate stakeholders (De Vreese et al. 2019), and misrepresent conflicts (Hattingh 2014). For example, as was shown in a case study about perceptions of the benefits from and threats to nature in Tierra del Fuego National Park in Argentina, assuming that stakeholders are only motivated by monetary gains does not correspond to the values expressed by the park's primary users and prevents environmental management to better align with public perceptions and needs (Mrotek et al. 2019).

In policymaking, relational values, as they are defined according to the proposed core meaning in the present article, can help articulate, as has emerged from the literature review, the idea that a specific place—a forest, a river, a landscape, or a population—are essentially important to people because of the unique relationships, history, and traditions that bind them together, as is expressed, for example, in the Japanese philosopher Watsuji Tetsurō’s concept of *fūdo* (風土), which refers to interrelationships between people and local characteristics (Prominski 2014). To date, relational values in policy documents primarily highlight targets and strategies rather than direct specific actions, but the academic literature suggests that they can benefit policies directly by accounting for contextual NCP (Díaz et al. 2018). Integrating relational values into policy actions can help operationalize broad policy guidance (e.g., IPBES) to regional, national, and local scales (Kitheka et al. 2019). Relational values can catalyze motivation and appeal to a broader audience (Stenseke 2018, Winkler and Hauck 2019), particularly for IPLC (Himes and Muraca 2018, Gould et al. 2019) and can increase the participation of different stakeholders (Jax et al. 2018, Kitheka et al. 2019). By stressing reciprocal relationships, they can facilitate social equity and environmental sustainability (Kenter et al. 2011, Diver et al. 2019). Although relational values can be assessed using sociocultural quantitative methods (Bryce et al. 2016, Schulz and Martin-Ortega 2018, Huynh et al. 2022), qualitative, participatory, and mixed methods approaches, as well as the employment of sociocultural indicators, more fully capture their meaning.

To summarize, from our literature review, it clearly emerges that each value type is critical for expressing some dimensions of why nature matters and that each value type can be a pragmatic leverage point for change, although the effectiveness of engaging with any single value type depends on the social, political, institutional, and ecological context. At the same time, each of these value types has limitations, but those limitations are often complimented by the strengths of other value types. For example, relational values and instrumental values can complement each other when used in tandem by highlighting trade-offs and potential synergies between financial costs and benefits and less easily quantified cultural, spiritual, and constitutive values. For this reason, we believe that a pluralistic approach to value assessment, values research, and value theory is the best path toward more just and sustainable solutions for nature and people.

In this context, recognizing the fuzzy boundaries between different values is necessary to implement value pluralism in practice. Being able to navigate this diversity, rather than use value types as static categories, can lead to more accurate outcomes in research and policy. In this sense, we believe that fuzzy boundaries are low-hanging fruit because their importance is already being expressed in terms of multiple value types. We suggest that engaging different stakeholders or groups with fuzzy boundaries between distinct expressions of value is likely a fruitful starting place to finding commonalities that can help mitigate conflict by making clear the plurality of values at play.

In the case of the aesthetic value, for example, different justifications might overlap and converge on the shared value of beauty, regardless of how each justification is articulated: a specific place, ecosystem, landscape, or experience can be considered beautiful by different groups of people for different reasons. Agreement on the aesthetic value can be a common starting point for dialogue, developing mutually agreed boundaries, or mediating across different social groups in support of conservation practices and policy.

At the same time, identifying specific articulation of value coming together in fuzzy boundaries can help identify lines of conflicts and take into account diverse knowledge and value systems. Although, for example, significant alliances across stakeholders might be constructed around the idea of life-support values, leaving space for diverse articulations of why and how they matter to different social groups in their own terms may point to underlying reasons for contention. For example, in the US Pacific Northwest, salmon are keystone species for ecologists and environmentalists, and they are foundational for the collective identity and the material and spiritual existence of many local tribes, facilitating alliances to restore waterways and protect salmon from imminent extinction (Salmon Orca Project 2023). With these benefits in mind, the diverse expressions of the importance of salmon are brought into fruitful coexistence toward common goals such as dam removal, but, because they are not conflated, they may also illuminate areas of contention that could undermine collaboration, such as the role of fish hatcheries (Fox et al. 2022) or the prioritization of tribal fishing rights. When considered in this way, research on life-support values can offer a potential common ground for encounters across different epistemic traditions and knowledge systems, within and beyond academia, in which diverse articulations can coexist and in which cross-fertilization is possible (Tengö et al. 2014).

### Study limitations

Systematic literature searches are limited by the databases and search string used to identify articles. Accordingly, the present article reflects a limited set of knowledge that neglects oral traditions, gray literature, and other forms of nonacademic knowledge. In addition, some key publications on relational values (Muraca 2011, Díaz et al. 2015, Chan et al. 2016) did not appear because the combination of value types and *nature, ecosystem services*, or *nature's contributions to people* did not occur in the title, abstract, keywords, or subject, even though they occurred in the text. For this reason, expert knowledge and consideration of additional sources was essential to contextualize, integrate, and interpret the results. Overall, the 239 coded papers augmented by the present authors’ knowledge of the literature are comprehensive of the current leading debates on specific environmental values.

Another limitation in our findings is the focus on English-language literature. A wider consideration of the semantic field of each value type (if not of the exact wording)—for example, in Spanish or Chinese—could offer other salient articulations and relevant associations. Moreover, the framework remains embedded in the Western traditions of environmental ethics, conservation biology, political ecology, and ecological economics. The search terms are not as commonly used by IPLC. To partially address this limitation, the results of a parallel correlated search on ILK literature, which also included literature in Spanish, were analyzed via qualitative interpretation of selected papers and on the ground of their relevance to developing core meanings or identifying salient articulations and relevant associations.

For instance, some important contributions from ILK and non-English literature include broader conceptualizations of instrumental values not limited to Western worldviews or reducible to means to human ends, as is described in our core meaning. These uses of instrumental values also extend to diverse worldviews, including pluricentrism, which were not relevant associations of instrumental values identified in our assessment of the literature (IPBES 2022). In these cases, the language of instrumental values can help articulate the importance for IPLC of access to and use of necessities such as wild food plants and animals (Ghorbani et al. 2012) but also the need for protection from them, as with the protection of crops from elephants in the Congo Basin (Ngouhouo Poufoun et al. 2016).

Similarly, for the semantic field of relational values, other examples emerged from a parallel search on ILK literature that were not immediately apparent from the coded literature. One reason is that in the case of relational ontologies or cosmovisions (Acuña et al. 2015, Escobar 2018, Diver et al. 2019), relational values are rarely articulated in the definitory language of *specific values* (as values of relationships between people and nature and among people through nature), although they also encompass and inform specific values with respect to contextual NCP and place-specific relations. In many cases, relational language is expressed with reference to general norms or instructions that guide practices (e.g., gathering, hunting, growing, ceremonies) and regulate use and access or principles that organize ways of life, modes of cohabitation with other-than-humans, obligations, and reciprocity (Singh 2013, Rahder 2014, Gould et al. 2019, Solís and Casas 2019). For example, the Cuicatec people in Mexico have rules associated with hunting and gathering seasons that respect female individuals of vertebrate species (Solís and Casas 2019). Similarly, the Monpa in Arunachal Pradesh, India, have environmental management practices emphasizing respectful land use influenced by traditional knowledge and the cultural network among community members (Singh 2013).

## Closing remarks

In the present article, we summarized the most frequent meanings and categorized heterogenous uses of intrinsic, instrumental, and relational values in the literature systematically into salient articulations and provided qualitative assessments of the strength of association between each value type and the worldviews described in the values assessment. We then explored fuzzy boundaries, where specific values overlap in the literature. Finally, we addressed how these results inform policy and can help direct future research.

We believe that having a clear understanding of the different value types and the ways they are used in the literature advances the potential for pluralistic valuation of ecosystem services and NCP and can inform better policy decisions. There is large consensus in the literature we reviewed that considering diverse values can help policymakers by making otherwise neglected, intangible costs and benefits visible (Witt et al. 2019), facilitate a more inclusive and just articulation of values (Himes and Muraca 2018), mitigate conflicts by fostering comanagement (Kenter et al. 2015, García-Llorente et al. 2018), and encourage participation and improve communication among different groups (Hope and Jones 2014, Reed and Ceno 2015, Arias-Arévalo et al. 2017, Berry et al. 2018, Gale and Ednie 2019, Witt et al. 2019). It can strengthen the motivations of people toward conservation (Winkler and Hauck 2019), enable better collaboration across disciplines (Chan et al. 2018), and support broad alliances for win–win solutions (Reyers et al. 2012). Pluralistic value assessments also reduce the risk of crowding out other motivations and help build common ground and reciprocal learning across different stakeholders by acknowledging different motivations (Rico García-Amado et al. 2013).

However, for theoretical consistency and accuracy in policy use, it is important to clarify the terminology regarding the different values at play in pluralistic assessments. Simultaneously, the fuzzy boundaries between values can indicate convergences that may be useful to build common ground across different groups in support of biodiversity conservation or equitable development (Norton 1991, Berry et al. 2018): “Environmentalists may consistently disagree over the reasons for a specific policy direction without disagreeing over the policy direction itself” (Saner and Bordt 2016, p. 76).

For future research, we believe the core meanings, salient articulations, and relevant associations we identified can help guide the development of survey instruments and coding of interview data in empirical studies on why nature matters. We also believe our framework can be used to develop approaches of ecosystem accounting that consider the multiple values of NCP, including ecosystem services. However, work should continue so that we can better understand the diversity of reasons that nature matters to people; broader linguistic articulations and nonlinguistic, embodied expressions related to intrinsic, instrumental, and relational values (and maybe other less characterized value types) are important to adequately represent worldviews and perspectives from cultures that do not share European philosophical history or publish in English. This would also support more explicitly investigating how different values can be expressed through diverse human–nature relationships. Increased inclusion of these diverse perspectives may clarify points of contention or confusion in management situations that can escalate to conflict.

## Author Biography

Austin Himes (ah3798@msstate.edu) is affiliated with the Department of Forestry and with the Forest and Wildlife Research Center, both at Mississippi State University, in Starkville, Mississippi, in the United States. Barbara Muraca is affiliated with the Department of Philosophy and the Environmental Studies Program at the University of Oregon, in Eugene, Oregon, in the United States. Christopher B. Anderson is affiliated with the Instituto de Ciencias Polares, Ambiente y Recursos Naturales at the Universidad Nacional de Tierra del Fuego and with the Centro Austral de Investigaciones Cientificas, in the Consejo Nacional de Investigaciones Científicas y Técnicas, both in Ushuaia, Tierra del Fuego, Argentina. Simone Athayde is affiliated with the Department of Global and Sociocultural Studies and the Kimberly Green Latin American and Caribbean Center, at Florida International University, in Miami, Florida, in the United States. Thomas Beery is affiliated with the School of Natural Science, Sustainable Multifunctional Landscapes, at Kristianstad University, in Kristianstad, Sweden. Mariana Cantú-Fernández is affiliated with the Instituto de Investigaciones en Ecosistemas y Sustentabilidad at the Universidad Nacional Autónoma de México, in Ciudad de Mexico, Mexico. David González-Jiménez is also affiliated with the Instituto de Investigaciones en Ecosistemas y Sustentabilidad at the Universidad Nacional Autónoma de México, in Ciudad de Mexico, and also with the Ceiba Centro de Formación y Desarrollo, in Oaxaca, Mexico. Rachelle K. Gould is affiliated with the Rubenstein School of Environment and Natural Resources at the University of Vermont, in Burlington, Vermont, in the United States. A. P. Hejnowicz is affiliated with the Global Change Institute, in the School of Geosciences at the University of Edinburgh, in Scotland, in the United Kingdom; with the School of Engineering at Newcastle University, in Newcastle upon Tyne, England, in the United Kingdom; and with the Department of Environment and Geography at the University of York, in York, England, in the United Kingdom. Jasper Kenter is affiliated with the Aberystwyth Business School, at Aberystwyth University, in Aberystwyth, Wales, in the United Kingdom; with Ecologos Research Ltd, also in Aberystwyth; and with the Department of Environment and Geography at the University of York, in York, England, in the United Kingdom. Dominic Lenzi is affiliated with the Department of Philosophy at the University of Twente, Enschede, in the Netherlands. Ranjini Murali is affiliated with the Geography Department at the Humboldt-Universitat zu Berlin, in Berlin, Germany, and with the Snow Leopard Trust, in Seattle, Washington, in the United States. Unai Pascual is affiliated with the Terrestrial Ecosystems Research Line at the Basque Centre for Climate Change, in Leioa, and with the Ikerbasque Basque Foundation for Science, in Bilbao, both in Spain, and with the Centre for Development and Environment at the University of Bern, in Bern, Switzerland. Christopher Raymond is affiliated with the Helsinki Institute of Sustainability Science; the Ecosystems and Environment Research Program, and the Department of Economics and Management at the University of Helsinki, in Helsinki, Finland. Annalie Ring is affiliated with the Department of Philosophy at the University of Oregon, in Eugene, Oregon, in the United States. Kurt Russo is affiliated with the intertribal nonprofit organization Se'Si'Le, in Eugene, Oregon, in the United States. Aibek Samakov is affiliated with the Hydro Nation International Centre, in Aberdeen, Scotland, in the United Kingdom. Sanna Stålhammar is affiliated with the Department of Landscape Architecture at the Swedish University of Agricultural Sciences, in Uppsala, Sweden. Henrik Thorén is affiliated with the Department of Philosophy at Lund University, in Lund, Sweden. Egleé Zent is affiliated with the Lab Ecología Humana at the Instituto Venezolano de Investigaciones Científicas, in Caracas, Distrito Capital, Venezuela.
