# Correction to: Why nature matters: A systematic review of intrinsic, instrumental, and relational values

**DOI:** 10.1093/biosci/biae024

**Published:** 2024-03-12

**Authors:** Austin Himes, Barbara Muraca, Christopher B Anderson, Simone Athayde, Thomas Beery, Mariana Cantú-Fernández, David González-Jiménez, Rachelle K Gould, A P Hejnowicz, Jasper Kenter, Dominic Lenzi, Ranjini Murali, Unai Pascual, Christopher Raymond, Annalie Ring, Kurt Russo, Aibek Samakov, Sanna Stålhammar, Henrik Thorén, Egleé Zent

**Affiliations:** Department of Forestry, Forest and Wildlife Research Center, Mississippi State University, Starkville, Mississippi, United States; Department of Philosophy and the Environmental Studies Program at the University of Oregon, Eugene, Oregon, United States; Instituto de Ciencias Polares, Ambiente y Recursos Naturales at the Universidad Nacional de Tierra del Fuego, Ushuaia, Tierra del Fuego, Argentina; Centro Austral de Investigaciones Cientificas, Consejo Nacional de Investigaciones Científicas y Técnicas, Ushuaia, Tierra del Fuego, Argentina; Department of Global and Sociocultural Studies; Kimberly Green Latin American and Caribbean Center at Florida International University, Miami, Florida, United States; School of Natural Science, Sustainable Multifunctional Landscapes at Kristianstad University, Kristianstad, Sweden; Instituto de Investigaciones en Ecosistemas y Sustentabilidad at the Universidad Nacional Autónoma de México, Ciudad de Mexico, Mexico; Instituto de Investigaciones en Ecosistemas y Sustentabilidad at the Universidad Nacional Autónoma de México, Ciudad de Mexico; Ceiba Centro de Formación y Desarrollo, Oaxaca, Mexico; Rubenstein School of Environment and Natural Resources at the University of Vermont, Burlington, Vermont, United States; Global Change Institute, School of Geosciences at the University of Edinburgh, Scotland, United Kingdom; School of Engineering at Newcastle University, Newcastle upon Tyne, England, United Kingdom; Department of Environment and Geography at the University of York, York, England, United Kingdom; Aberystwyth Business School, at Aberystwyth University, Aberystwyth, Wales, United Kingdom; Ecologos Research Ltd, Aberystwyth; Department of Environment and Geography at the University of York, York, England, United Kingdom; Department of Philosophy at the University of Twente, Enschede, the Netherlands; Geography Department at the Humboldt-Universitat zu Berlin, Berlin, Germany; Snow Leopard Trust, Seattle, Washington, United States; Terrestrial Ecosystems Research Line at the Basque Centre for Climate Change, LeioaSpain; Ikerbasque Basque Foundation for Science, Bilbao, Spain; Centre for Development and Environment at the University of Bern, Bern, Switzerland; Helsinki Institute of Sustainability Science; the Ecosystems and Environment Research Program; Department of Economics and Management at the University of Helsinki, Helsinki, Finland; Department of Philosophy at the University of Oregon, Eugene, Oregon, United States; intertribal nonprofit organization Se'Si'Le, Eugene, Oregon, United States; Hydro Nation International Centre, Aberdeen, Scotland, United Kingdom; Department of Landscape Architecture at the Swedish University of Agricultural Sciences, Uppsala, Sweden; Department of Philosophy at Lund University, Lund, Sweden; Lab Ecología Humana at the Instituto Venezolano de Investigaciones Científicas, Caracas, Distrito Capital, Venezuela

This is a correction to: Austin Himes, Barbara Muraca, Christopher B Anderson, Simone Athayde, Thomas Beery, Mariana Cantú-Fernández, David González-Jiménez, Rachelle K Gould, A P Hejnowicz, Jasper Kenter, Dominic Lenzi, Ranjini Murali, Unai Pascual, Christopher Raymond, Annalie Ring, Kurt Russo, Aibek Samakov, Sanna Stålhammar, Henrik Thorén, Egleé Zent, Why nature matters: A systematic review of intrinsic, instrumental, and relational values, *BioScience*, Volume 74, Issue 1, January 2024, Pages 25–43, https://doi.org/10.1093/biosci/biad109

In the original publication of the manuscript, there was a typographical error in the name of the 19th author. This should read: “Henrik Thorén.”

The emendation has been made in the article.

## Author Biography

Austin Himes (ah3798@msstate.edu) is affiliated with the Department of Forestry and with the Forest and Wildlife Research Center, both at Mississippi State University, in Starkville, Mississippi, in the United States. Barbara Muraca is affiliated with the Department of Philosophy and the Environmental Studies Program at the University of Oregon, in Eugene, Oregon, in the United States. Christopher B. Anderson is affiliated with the Instituto de Ciencias Polares, Ambiente y Recursos Naturales at the Universidad Nacional de Tierra del Fuego and with the Centro Austral de Investigaciones Cientificas, in the Consejo Nacional de Investigaciones Científicas y Técnicas, both in Ushuaia, Tierra del Fuego, Argentina. Simone Athayde is affiliated with the Department of Global and Sociocultural Studies and the Kimberly Green Latin American and Caribbean Center, at Florida International University, in Miami, Florida, in the United States. Thomas Beery is affiliated with the School of Natural Science, Sustainable Multifunctional Landscapes, at Kristianstad University, in Kristianstad, Sweden. Mariana Cantú-Fernández is affiliated with the Instituto de Investigaciones en Ecosistemas y Sustentabilidad at the Universidad Nacional Autónoma de México, in Ciudad de Mexico, Mexico. David González-Jiménez is also affiliated with the Instituto de Investigaciones en Ecosistemas y Sustentabilidad at the Universidad Nacional Autónoma de México, in Ciudad de Mexico, and also with the Ceiba Centro de Formación y Desarrollo, in Oaxaca, Mexico. Rachelle K. Gould is affiliated with the Rubenstein School of Environment and Natural Resources at the University of Vermont, in Burlington, Vermont, in the United States. A. P. Hejnowicz is affiliated with the Global Change Institute, in the School of Geosciences at the University of Edinburgh, in Scotland, in the United Kingdom; with the School of Engineering at Newcastle University, in Newcastle upon Tyne, England, in the United Kingdom; and with the Department of Environment and Geography at the University of York, in York, England, in the United Kingdom. Jasper Kenter is affiliated with the Aberystwyth Business School, at Aberystwyth University, in Aberystwyth, Wales, in the United Kingdom; with Ecologos Research Ltd, also in Aberystwyth; and with the Department of Environment and Geography at the University of York, in York, England, in the United Kingdom. Dominic Lenzi is affiliated with the Department of Philosophy at the University of Twente, Enschede, in the Netherlands. Ranjini Murali is affiliated with the Geography Department at the Humboldt-Universitat zu Berlin, in Berlin, Germany, and with the Snow Leopard Trust, in Seattle, Washington, in the United States. Unai Pascual is affiliated with the Terrestrial Ecosystems Research Line at the Basque Centre for Climate Change, in Leioa, and with the Ikerbasque Basque Foundation for Science, in Bilbao, both in Spain, and with the Centre for Development and Environment at the University of Bern, in Bern, Switzerland. Christopher Raymond is affiliated with the Helsinki Institute of Sustainability Science; the Ecosystems and Environment Research Program, and the Department of Economics and Management at the University of Helsinki, in Helsinki, Finland. Annalie Ring is affiliated with the Department of Philosophy at the University of Oregon, in Eugene, Oregon, in the United States. Kurt Russo is affiliated with the intertribal nonprofit organization Se'Si'Le, in Eugene, Oregon, in the United States. Aibek Samakov is affiliated with the Hydro Nation International Centre, in Aberdeen, Scotland, in the United Kingdom. Sanna Stålhammar is affiliated with the Department of Landscape Architecture at the Swedish University of Agricultural Sciences, in Uppsala, Sweden. Henrik Thorén is affiliated with the Department of Philosophy at Lund University, in Lund, Sweden. Egleé Zent is affiliated with the Lab Ecología Humana at the Instituto Venezolano de Investigaciones Científicas, in Caracas, Distrito Capital, Venezuela.

